# Remote Effects of Chloroform

**Published:** 1856-06

**Authors:** 


### *Remote Effects of Chloroform.*

We take the following interesting facts, in relation to a very interesting subject, from an article in the *Charleston Medical Journal*, by Dr. C. Happoldt, the Editor:—

"Besides showing the large quantity of chloroform which may be introduced into the system without producing death, I am desirous of calling attention, in the following remarks, to some of its local effects on the nerves of special sense—whose seat of function is located in the passages through which the vapor passes on its way to the lungs—and to its remote effects produced on the organs supplied by the nerves which proceed from the lower segment of the spinal column.

"Two cases recently came under my observation, which are interesting for the exhibition of these phenomena. The subjects of both were of the nervo-lymphatic temperament, and of sedentary habits. They had, during several months, resorted to the inhalation of chloroform for the purpose of procuring sleep, when this desired condition did not naturally occur at a seasonable hour. One or two ounces generally induced tranquil slumber, without being followed by any unpleasant consequence, except slight nausea, which usually dissappeared after the morning meal.

"One of these patients had an attack of asthma, to which he had been previously subject; and being aware of the anti-spasmodic and anæsthetic properties of chloroform, he unadvisably put himself under its influence, and continued the inhalation for forty hours; during which time he inhaled *twenty ounces of the fluid.* The asthma was relieved, and has not since returned—nine months having now elapsed—but he was left in an uncomfortable condition: the sense of smell was abolished, and that of taste perverted. The bladder and rectum lost their tonicity and excitability: when the former became distended, a concentrated effort of the will was necessary to effect urination; the latter remained for several months in a torpid condition, requiring the constant use of cathartics to effect the evacuation of its contents. The sexual appetite was, for many weeks, abolished; and the restoration of the functions of these organs was slowly accomplished. Saline substances were urgently craved, and freely taken. Brandy was not disagreeable, and appeared to be of use in restoring the healthy conditions of the organs involved.

"The other patient supposed that he inhaled four fluid ounces without interruption, from the fact, that the phial which had contained that quantity was empty on the following morning. He remained unconscious ten hours, and, on awakening, experienced no unpleasant symptoms. While breakfasting, he noticed the strange taste of the various dishes of which he partook; but the taste of coffee was peculiarly unpleasant; and it was with difficulty that he could be persuaded that it was not the substances which he ate, but his sense of taste, which was at fault. During the day, whatever he ate appeared perverted in flavor; he became convinced that the cause of his altered sensations lay in his organ of taste. For more than a month, neither fruits, wines, tea, nor coffee, could be taken with relish; and it was not until the expiration of two months that the sense of taste was restored.

"The sense of smell was, for nearly the same length of time, almost abolished. The nostrils were nearly closed by the swelling of the mucous membrane. The tongue became pale in color; and the mucus membrane of the mouth and throat was flaccid and swollen, as was that of the nares.

"Coincident with these phenomena, the penis was felt to be unusually flaccid; and there was no inclination to urinate. It was only after the bladder became considerably distended that urination was possible. There was no pain, but a strange sensation along the uretha while the urine was passing out. The specific gravity of this excretion was somewhat below the normal standard, and it contained a large proportion of the triple phosphates, and a trace of the crystals of uric acid. During two months there was no erection of the penis. The patient believed that the secretion of semen was not interfered with, from the sensation referred to the testes, and the desire which existed for sexual indulgence. The inability to perform the act he attributed solely to the paralysis of the perineal muscles.

"The rectum and intestines partook of a similar torpidity with the urinary organs; but the sphincter ani retained its contractile power. For a week there was no evacuation from the bowels, and no uneasiness was felt therefrom. The saline cathartics had very little effect. Calomel, rhubarb, aloes, and other more drastic substances, were found most efficacious. Nux vomica and strychnine, arnica, and iron, were resorted to, with no perceptible effect over the paralyzed organs. Brandy, which was most agreeable to the taste as a beverage, appeared to mitigate the symptoms, and was freely taken during their continuance.

"After the expiration of two months, the only remaining effect of the chloroform was constipation, which remains until this time (December 20th,) five months since the last inhalation. This patient had never before suffered from constipation; now defecation seldom occurs without the aid of a cathartic."

